# Distinct gastric phenotype in patients with pathogenic variants in *SMAD4:* A nationwide cross-sectional study

**DOI:** 10.1055/a-1954-0522

**Published:** 2022-12-15

**Authors:** Anne Marie Jelsig, Niels Qvist, Birgitte Bertelsen, Lise-Lotte Christensen, Hanne Grossjohan, Charlotte Kvist Lautrup, Lone Sunde, Pernille Mathiesen Tørring, Ken Ljungman, Louise Torp Christensen, John Gásdal Karstensen

**Affiliations:** 1 Department of Clinical Genetics, University Hospital of Copenhagen, Rigshospitalet, Denmark; 2 Research Unit for Surgery, Odense University Hospital, Odense Denmark; University of Southern Denmark, Odense, Denmark; 3 Department of Genomic Medicine, Copenhagen University Hospital, Rigshospitalet, Denmark; 4 Department of Molecular Medicine, University Hospital of Aarhus, Denmark; 5 Department of Gastrointestinal Surgery, University Hospital of Copenhagen, Rigshospitalet, Denmark; 6 Department of Clinical Genetics, Aarhus University Hospital, Aarhus, Denmark; 7 Department of Clinical Genetics, Aalborg University Hospital, Denmark; 8 Department of Clinical Genetics, Odense University Hospital, Denmark; 9 Department of Surgery, Aarhus University Hospital, Arhus, Denmark; 10 Department of Surgery, Slagelse Hospital, Slagelse, Denmark; 11 Danish Polyposis Registry, Gastrounit, Copenhagen University Hospital – Amager and Hvidovre, Hvidovre, Denmark; 12 Department of Clinical Medicine, University of Copenhagen, Denmark

## Abstract

**Background and study aims** In most patients with juvenile polyposis Syndrome, it is possible to detect a pathogenic germline variant in *SMAD4* or *BMPR1A* . It is well known that patients with a pathogenic variant in *SMAD4* have a higher risk of gastric polyposis and gastric cancer compared to *BMPR1A* carriers, but the natural history of gastric involvement is poorly described. We aimed to systematically review endoscopic and histopathological gastric findings in Danish patients with pathogenic variants in *SMAD4.*

**Patients and methods** This was a retrospective, cross-sectional study including endoscopic and histological gastric findings in all known Danish patients with pathogenic variants in *SMAD4* . The patients were identified by data from various registries as well as from clinical genetic departments and laboratories.

**Results** We identified 41 patients (2–72 years) with a pathogenic *SMAD4* variant *.* In 31 patients, we were able to retrieve information on upper gastrointestinal endoscopy. Eighty-seven percent had at least one gastric abnormality including erythema (72 %) and edema (72 %). Half of the patients also had vulnerability of the mucosa and 68 % had gastric polyposis. An increasing frequency of abnormalities were observed with increasing age. Gastric cancer was diagnosed in 5 % of the cases and 22 % had a gastrectomy mainly because of massive polyposis.

**Conclusions** This study showed that most patients with pathogenic *SMAD4* variants have a distinct phenotype of the gastric mucosa, and with an increasing severity in the elderly patients. These findings provide new insights into the natural history of gastric manifestations in patients with pathogenic *SMAD4* variants.

## Introduction

Juvenile polyposis syndrome (JPS, OMIM 174900) is a rare hereditary disorder characterized by the occurrence of often multiple juvenile polyps in the gastrointestinal tract 1] Introduction, mainly in the colon and stomach. In most patients with JPS, a pathogenic germline variant can be detected in either *SMAD4* (1/3) or *BMPR1A* (1/3). As patients with pathogenic variants (PV) in *SMAD4* often have additional symptoms of Hereditary Haemorrhagic Telangiectasia (JP-HHT syndrome) and increased risk of thoracic aortic aneurisms, a multidisciplinary follow-up from an early age is recommended 1 2 . The expressivity of JPS is variable, but most patients with a PV in *SMAD4* will during their lifetime fulfill the clinical criteria of JPS (more than five colorectal juvenile polyps) 3 .

JPS patients have an increased lifetime risk of both colorectal cancer (CRC) and gastric cancer (GC), and it is well-documented that gastric polyposis and GC are far more common in carriers of a PV in *SMAD4* than in *BMPR1A* variant carriers 3 4 5 . Massive gastric polyposis, as seen in patients with PVs in *SMAD4* , can make surveillance with gastroesophageal endoscopy difficult or result in severe obstructive symptoms and gastrectomy might be indicated. However, the natural history of gastric involvement as well as threshold for gastrectomy are poorly described. To increase the understanding about gastric affection in patients with a PV in *SMAD4* , we systematically reviewed endoscopic and histopathological gastric findings in Danish patients.

## Patients and methods

The study was a nationwide cross-sectional study approved by The Danish Patient Safety Authority (journal no. 31–1521–329) and the Regional Danish Data Protection Agency (journal no.: P-2020–696, Capital Region of Denmark).

### Patient identification

All Danish genetic departments and laboratories were asked to identify patients with a PV in *SMAD4* . If the department had information about affected relatives, they were included as well if they carried the variant. Data were also retrieved from The Danish Pathology Register, which comprises all histopathological examinations carried out in Denmark since the mid-1990 s, and for some parts of Denmark even earlier. A search was performed using the Danish version of the Systematized Nomenclature of Medicine (SNOMED) diagnostic codes for “juvenile polyp” and “hamartomatous polyp.” The terms were combined with the word “polyposis.” Patients fulfilling the clinical diagnostic criteria for JPS (more than 5 colorectal juvenile polyps) were noted and their medical files were searched for whether a PV in *SMAD4* had been detected. A patient was included if he/she was heterozygous for a PV (a variant classified as *pathogenic* or *likely pathogenic* according to the American College of Medical Genetics (ACMG) criteria detected in the laboratories 6 ). Both alive and deceased patients of all ages were included.

### Data collection

Data from all relevant departments were collected and analyzed. Gastric findings were evaluated by inspecting endoscopic images when available and by the endoscopic description and histopathological results. All results/images were evaluated by a gastrointestinal endoscopist with expert knowledge of upper gastrointestinal endoscopy (JGK) and in most cases also by the local surgeon. They noted and evaluated whether the gastric mucosa was being “erythematous,” “edematous,” “vulnerable” and whether “polyposis” and/or hiatal hernia were present. The term “vulnerability” was defined as bleeding when the mucosa was touched by the endoscope and/or a biopsy forceps. For patients who had a gastrectomy, the histopathological reports were retrieved. We also searched for the histopathological reports of biopsied material and removed polyps.

### Statistics

The point prevalence for 2021 was calculated based on the total Danish population retrieved from Statistics Denmark (5,843,347 residents). Data are presented in absolute numbers ( Table 1 and Table 2 ) and proportion (%). Differences between groups were compared with a χ ^2^ -test for categorical variables. *P =* 0.05 defined the level of significance. All statistics were performed using SAS enterprise Guide (Version 7.1; SAS Institute Inc., Cary, North Carolina, United States).

**Table 1:** Characteristics of 41 patients carrying a pathogenic variant in *SMAD4.*

	**Findings**
No. of patients (age range) at study inclusion	41 (2–78y)
Female:male	20:21
No. of families	15
No. of patients deceased (mean age at death)	9 (54y)
Origin of gene variant: De novo;inherited;unknown	7:5:3
**Gastric finding**	
No. of patients for which information about endoscopy was available	31
No. of patients for which detailed description and/or images of the gastric ventricle where available	29
No. of patients with at least one abnormal gastric finding	27/31 (87 %)
No. of patients with gastric polyposis (all)	21/31 (68 %)
Local polyposis	4/29 (7 %)
Diffuse polyposis	16/29 (55 %)
No. of patients who had had gastric cancer (mean age)	2/41 (42)
No. of patients who had had gastrectomy without gastric cancer (mean age at operation)	9/41 (44y)
**Gastric mucosa**	
Edema	21/29 (72 %)
Vulnerability	15/29 (52 %)
Erythema	21/29 (72 %)
Other	
Hiatal hernia	9/29 (31 %)

**Table 2:** Gastric findings in patients with a pathogenic variant in SMAD4.

	**15–20 years**	**20–29 years**	**30–39 years**	**40–49 years**	**50–59 years**	**60–69 years**	**70 + years**	***P* value **
Edema	0/1	2/6 (33 %)	6/8 (75 %)	6/6 (100 %)	5/5 (100 %)	2/2 (100 %)	0/1	0.022
Vulnerability	0/1	0/6 (0 %)	4/8 (50 %)	4/6 (66 %)	5/5 (100 %)	2/2 (100 %)	0/1	0.016
Erythema	0/1	4/6 (66 %)	5/8 (63 %)	6/6 (100 %)	4/5 (80 %)	2/2 (100 %)	0/1	0.177
Polyposis	0/1	1/6 (17 %)	6/8 (75 %)	5/6 (83 %)	4/5 (80 %)	2/2 (100 %)	1/1	0.076
Localized polyposis	0/1	1/6 (17 %)	2/8 (25 %)	1/6 (17 %)	0/5 (0 %)	0/2 (0 %)	1/1	0.339
Diffuse polyposis	0/1	0/6(0 %)	4/8 (50 %)	4/6 (67 %)	4/5 (80 %)	2/2 (100 %)	0/1	0.053

## Results

### Patient characteristics

The total number of patients identified with a PV in *SMAD4* was 41 (females: 20) comprising 15 families. The mean age of patients at the time of data retrieval was 39.9 years (2–72) with four patients being under 18 years of age. Thirty-two patients were alive. The prevalence of carriers of a PV in *SMAD4* in the Danish population is app. 1:200,000.

The PVs in *SMAD4* included frameshift, missense, and nonsense variants (Supplementary Table 1). In addition, a balanced reciprocal chromosome translocation t(1;18)(p36.1;q21.1) with a breakpoint in *SMAD4* was detected in one family.

### Causes of death

Nine patients with *SMAD4* PVs were deceased. The mean age at death was 54 years (25–77). Two patients experienced sudden death of unknown causes, one before age 30 and the other before age 60 years; another two died of lung cancer, both under age 60 years. One patient died of GC and one died following complications to a thoracic aneurysm repair, both under age 40 years. The last three patients died of unknown causes at over 60 years of age.

### Endoscopic documentation

In 29 of 41 patients with a PV in *SMAD4,* it was possible to retrieve detailed information and/or endoscopic images that were sufficient for evaluating the gastric mucosa for all parameters (vulnerability, edema, erythema, and polyposis). In two patients, the patient record stated the presence of gastric polyposis that was not otherwise documented. In 10 patients, we were not able to find sufficient endoscopy reports or images: Four patients were under age 15 years, which is the age at which surveillance of the upper gastrointestinal tract begins in Denmark. In four patients, a post mortem diagnosis was made and data on it could not be retrieved (died before 1970) and one patient declined surveillance. In one patient, it was not possible to find a description nor endoscopic images; however, the patient had GC.

### Gastric findings

**Fig. 1:**
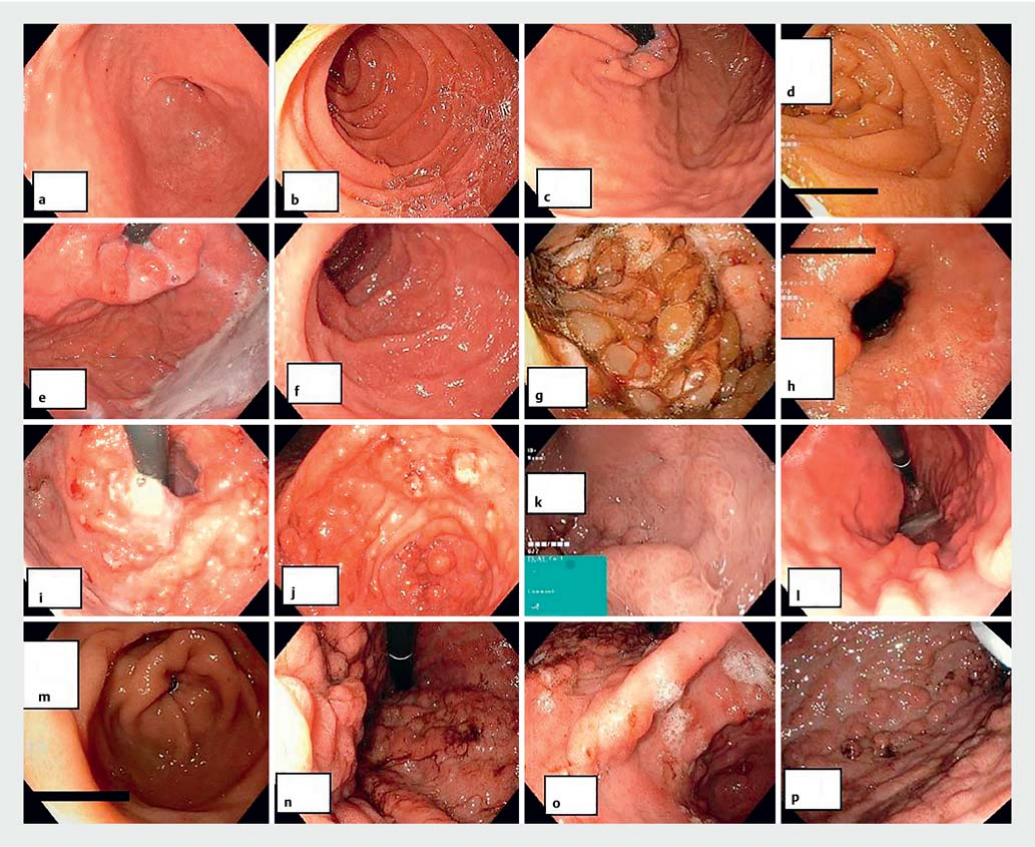
Endoscopic images from carriers of pathogenic *SMAD4* variants. **a, b, c, d** Patients in their twenties presenting with erythema. **e, f, g, h** Patients in their thirties with more severe presentations. **g** Patient no.22 before gastrectomy. **i, j, k, l** Patients in their forties. **i,j** Patient no.36 before gastrectomy (47y). **l** Patient no. 11 with a less severe gastric phenotype. **m, n, o, p** Patients in their 50. **n, o, p** Patient no.31 before gastrectomy.

Out of the 31 patients for which we had information, 27 had at least one gastric abnormality including erythema (72 %) and edema (72 %) of the gastric mucosa. Fifty-two percent of the patients also had vulnerability of the mucosa and 68 % of patients, had gastric polyposis. GC was diagnosed in 5 % of the patients and 22 % had a gastrectomy due to widespread polyposis and/or obstructive symptoms. Endoscopic images are depicted in Fig. 1 . Four patients (13 %) had no abnormal gastric findings, and they were all under 35 years of age at the time of gastroscopy.

### Correlation with age

**Fig. 2:**
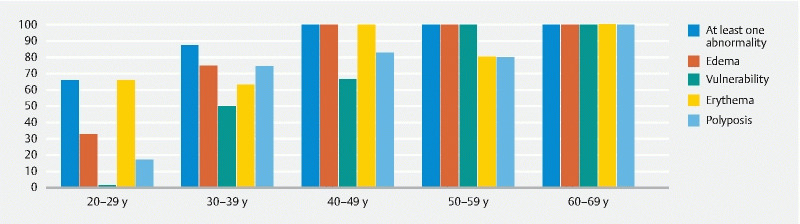
Abnormal gastric findings according to age in carriers of a pathogenic variant in *SMAD4* . The percentage is shown on the Y-axis.

The age of the patients at the most recent gastroesophageal endoscopy was noted, and accordingly, each patient was allocated to a decade as seen in Table 2 and Fig. 2 . As demonstrated, the age at which abnormalities were detected varied, but some trends are notable: Erythema, edema, and vulnerability were not present in the only patient younger than 20 years, while 33 % of patients aged 20 to 29 years had edema and 66 % had erythema. None in this age group had a vulnerable mucosa and only one patient had localized polyposis, while none had diffuse polyposis. In patients aged 30 to 39 years, 88 % had at least one gastric abnormality and 46 % had diffuse polyposis. All patients aged 40 to 49 years had at least one gastric abnormality, and diffuse polyposis and a vulnerable mucosa were present in 67 %. All patients aged 50 to 59 years had edema and a vulnerable mucosa, while 80 % had diffuse polyposis, whereas none had localized polyposis. All patients aged 50 to 59 years had edema and a vulnerable mucosa and 80 % had diffuse polyposis and erythema. Two patients aged 60 to 69 years presented with all abnormalities. The only significant differences between the age groups were in edema and vulnerability ( Table 2 ).

### Gastrectomies and GC

A total of nine patients with a PV in S *MAD4* had gastrectomies (22 %) without detected GC; the youngest being approximately 30 years of age and the oldest approximately 60 years old at the time of surgery. In all patients, the indication for the gastrectomy was massive polyposis with obstructive symptoms and dysphagia with the dominant symptoms. In two families, at least two family members had a gastrectomy due to polyposis, whereas five patients had no relatives with a history of gastrectomy or GC.

Two patients (5 %) were diagnosed with GC ate under 50 years of age and had a gastrectomy and partial gastrectomy performed, respectively. One of these patients had no relatives with the proven *SMAD4* variant, while the other patient had five relatives who carried the *SMAD4* variant, none of whom were diagnosed with GC or had had a gastrectomy.

### Histopathology

In 10 of the 11 patients who had a gastrectomy performed and/or were diagnosed with GC, it was possible to find a rather detailed histopathological description. In all patients without cancer, severe polyposis covering all parts of the gastric mucosa from pylorus to the gastroesophageal junction was noted – except in one patient in whom the antrum was spared. In patients who had a gastrectomy because of massive polyposis, the polyposis was described as predominantly foveolar hyperplasia (n = 6), hyperplastic (n = 2), or hamartomatous (n = 1). Inflammation and edema of the lamina propria were found in seven patients. Dysplasia was detected in three cases: Low-grade dysplasia in a polyp in one patient and several areas of high-grade dysplasia in another patient – both aged 50 to 60 years. High-grade dysplasia was also found in a patient (> 60 years of age) with a large gastric polyp. The two patients who had GC were both diagnosed at under age 50 years. In the first patient, GC was found during surveillance. The tumor was localized in the cardiac area and described as a diffuse adenocarcinoma with signet ring cells. Although the patient received a partial gastrectomy, he died 33 months later due to metastatic disease. The other patient lived 20 years before she died of other causes; however, details of histopathology could not be retrieved. The descriptions of the organization, shape and color of the polyps were not consistent, and we were not able to derive systematic information regarding these features.

## Discussion

In this population, we found gastric alterations in 87 % of patients (27/31) with a PV in *SMAD4* . The abnormalities tended to be more extensive in older than in younger patients. The gastric mucosa was predominantly normal in younger patients; however, among those aged 20 to 29 years, about half already had erythematous, vulnerable and edematous mucosa, in some cases with localized polyposis. These abnormalities were present in almost all patients older than age 40 years. The frequency of diffuse polyposis was also higher in older patients than in younger patients, with some having had gastrectomy at a young age (30 to 40 years) due to massive polyposis with dysphagia and/or obstructive symptoms. The differences between the age groups were statistically significance regarding edema and vulnerability, but not for other features. However, the small number of patients are likely to affect the results.

We observed a variation in severity of the phenotype that was observed not only between families, but also within families. Thus, the presence of GC or other severe gastric manifestations in affected family members may not be very informative in relation to prediction of gastric manifestations in each patient. Although our data are cross-sectional and, therefore, do not document development of abnormalities over time, we did observe increasing frequency and severity of gastric abnormalities with increasing age. The four patients who did not have abnormalities were all under 35 years of age. If progression with age is confirmed in prospective studies, this would enable improved decision-making regarding continued endoscopic surveillance or surgery – similar to the Spigelman classification that is used for familial adenomatous polyposis (FAP) 7 8 .

According to the present Danish guidelines, all patients with JPS – no matter genotype – are recommended to participate a surveillance program with both gastroscopy and colonoscopy beginning at ages 12 to 15 9 . In addition, carriers of PVs in *SMAD4* are recommended to undergo thoracic echocardiography beginning in infancy and then every 5 years, as well as HHT-surveillance beginning at age 12 years (before age 12 if suspected HHT symptoms). Overall, gastric abnormalities were found in 90 % of carriers of PVs in *SMAD4* and our study also confirmed that gastric polyposis is common, because localized or diffuse polyposis was present in 70 % of patients, which is consistent with observations in other studies 3 5 10 11 . We demonstrated that most patients with PVs in *SMAD4* have a distinct signature of gastric manifestations, which may progress in severity over time beginning with erythema, edema and a vulnerable mucosa followed by polyposis and eventually GC. These findings make patient information on this matter important and gastric surveillance crucial 3 4 5 . Initiating surveillance at age 12 to 15 seems reasonable because abnormalities before that age were rare, although we should bear in mind that there are no long-term follow-up studies on the effect of gastric surveillance.

Among 41 Danish carriers of PVs in *SMAD4* , only two patients were diagnosed with GC. However, nine of the 32 patients who were alive were under age 30 years and 16 were under age 40 years. Furthermore, nine patients had a gastrectomy due to dysphagia and obstructive symptoms without having developed GC; therefore, the “natural” risk of GC may by substantially higher. The exact risk of GC in JPS varies in literature. Howe et al. 12 found that 21 % of JPS kindred had cancer in the upper gastrointestinal tract including GC, while Blatter et al. 3 reported four cases in 126 patients with JPS 3 12 . As with other cancer predispositions syndromes, GC tend to develop earlier than in sporadic cases. In the study by Blatter et al *.* the median age was 44 3 and in our study, both patients were under age 50 years.

Gastric abnormalities are reported in other hereditary polyposis syndromes including FAP, in which fundic gland polyposis is detected in most patients 13 . Gastric adenocarcinoma and proximal polyposis of the stomach (GAPPS) is characterized by gastric fundic gland polyposis and increased risk of GC but without colonic involvement 14 . The occurrence of fundic gland polyposis is also related to non-hereditary factors, including the use of proton pump inhibitors, making diagnostics difficult in some cases. Based on this study, we propose that patients with a PV in *SMAD4* have a distinct gastric phenotype, but these findings cannot stand alone when evaluating whether a patient has a hereditary polyposis syndrome. Patients with a hereditary polyposis syndrome often have colorectal adenomatous as seen in FAP or hamartomatous polyposis, a positive family history, as well as extraintestinal features. Genetic analysis with a panel of polyposis-related genes including the promotor of exon1B of *APC* (affected in GAPPS) will aid diagnostics and (mostly) determine the precise diagnosis.

It is not known why carriers of PVs in *SMAD4* have a greater risk of gastric manifestations and GC compared to *BMPR1A* variant carries or to patients with JPS without known etiology. SMAD4 is a central protein in the TGF-beta pathway, where SMAD4 mediates translocation of other SMAD proteins into the nucleus to activate transcription 15 . The central function of the protein may be the explanation for the rather complex phenotype with both HHT manifestations, thoracic aneurisms, and gastrointestinal manifestations compared to the phenotype in carriers of PVs in *BMPR1A* who, based on the current knowledge, mainly have manifestation in the lower GI-tract. It is also unclear what drives the development of polyps and cancer. The results from analysis for loss of heterozygosity of *SMAD4* in polyps and carcinomas have been inconsistent 16 17 18 . One could speculate that at least the erythema is part of the HHT-phenotype where the blood vessels are affected. But whether this and the edema somehow predisposes to polyposis and eventually cancer remains to be elucidated.

The strengths of our study are that we included patients systematically and nationwide in Denmark, in which all citizens are identifiable by a unique identifier in the Central Person Register, in which the health care system is public, and where reporting of health data to various national registers is mandatory. However, the number of patients in our study remains limited and the results may reflect ascertainment bias as patients are more likely to have genetic testing performed if they have severe symptoms and a family history of polyposis and/or cancer. Furthermore, there may be an interobserver disagreement when describing the gastric mucosa both between endoscopists but also between the pathologists. The interobserver variability needs to be elucidated in future studies as recently carried out for the Spigelman classification 6 . In addition, we performed a cross-sectional study, making it hard to derive risk estimates from the observed frequencies. Also, the level of details in the endoscopic and histopathological descriptions varied, making it difficult to obtain consistent information such as on *Helicobacter pylori* infection, shape, size and localization of gastric polyps. To obtain more valid information on risks, larger follow-up studies should be performed, and because few persons are carriers of a PV in *SMAD4* , this calls for international collaboration.

## Conclusions

This nationwide study including all Danish patients with PVs in *SMAD4* demonstrated a distinct signature of gastric manifestations. Our results indicate that the abnormalities may progress in severity over time, beginning with erythema, edema, and a vulnerable mucosa followed by polyposis and eventually GC. However, systematic prospective studies are necessary to confirm this hypothesis.

## Supplementary material

Supplementary material Zusatzmaterial
