# Supplementary material for: Distinct gastric phenotype in patients with pathogenic variants in SMAD4: A nationwide cross-sectional study
Source: Endosc Int Open. 2022 Dec 15;10(12):E1537–43. doi: 10.1055/a-1954-0522 (PMC9754866; doi:10.1055/a-1954-0522)
Supplement: Supplementary file 1 — Supplementary material [file 2768supmat_10-1055-a-1954-0522.pdf]

Supplementary material

Supplementary Table 1

Predisposing variants in *SMAD4*

Family nr.	Variant[1]		Classification[2]	Exon number[1]	Type
I	c.1156G>A	p.(Gly386Ser)	Likely pathogenic	Exon 10	Missense
II	t(1;18)(p36.1;q21.1)				Chromosomal translocation
III	c.723_730del	p.(Gly243Alafs*18)	Pathogenic	Exon 6	Frameshift
IV	c.1325_1326del	p.(Gln442Leufs*51)	Pathogenic	Exon 11	Frameshift
V	c.1245_1248del	p.(Asp415Glufs*20)	Pathogenic	Exon 10	Frameshift
VI	c.1421del	p.(Ser474Ter)	Pathogenic	Exon 11	Nonsense
VII	del (c.955+1_956-1)_(1308+1_1309-1)del)	p.(Ala319Glyfs*3)	Pathogenic	Exon 9-10	Frameshift
VIII	c.330dupA	p.(His111Thrfs*3)	Pathogenic	Exon 3	Frameshift
IX	c.1081C>T	p.(Arg361Cys)	Pathogenic	Exon 9	Missense
X	c.1245_1248del	p.(Asp415Glufs*20)	Pathogenic	Exon 10	Frameshift
XI	c.1587dup	p.(His530Thrfs*47)	Pathogenic	Exon 12	Frameshift
XII	c.939del	p.(Ile314Phefs*22)	Pathogenic	Exon 8	Frameshift
XIII	c.692dup	p.(Ser232Glnfs*3)	Pathogenic	Exon 6	Frameshift
XIV	c.1448-1G >A	p.?	Likely pathogenic	Intron 11	Splice site
XV	c.831_832del	p.(Pro278Ter)	Pathogenic	Exon 7	Nonsense

1. NM_005359.5
2. According to ACMG standards and guidelines (*Richards et al*. 2015)
